# GC-Rich Sequence Elements Recruit PRC2 in Mammalian ES Cells

**DOI:** 10.1371/journal.pgen.1001244

**Published:** 2010-12-09

**Authors:** Eric M. Mendenhall, Richard P. Koche, Thanh Truong, Vicky W. Zhou, Biju Issac, Andrew S. Chi, Manching Ku, Bradley E. Bernstein

**Affiliations:** 1 Howard Hughes Medical Institute and Department of Pathology, Massachusetts General Hospital and Harvard Medical School, Boston, Massachusetts, United States of America; 2 Center for Systems Biology and Center for Cancer Research, Massachusetts General Hospital, Boston, Massachusetts, United States of America; 3 Broad Institute of Harvard and Massachusetts Institute of Technology, Cambridge, Massachusetts, United States of America; 4 Division of Health Sciences and Technology, Massachusetts Institute of Technology, Cambridge, Massachusetts, United States of America; 5 Biological and Biomedical Sciences, Harvard Medical School, Boston, Massachusetts, United States of America; 6 Neuro-Oncology Division, Department of Neurology, Massachusetts General Hospital, Boston, Massachusetts, United States of America; University of California San Francisco, United States of America

## Abstract

Polycomb proteins are epigenetic regulators that localize to developmental loci in the early embryo where they mediate lineage-specific gene repression. In *Drosophila*, these repressors are recruited to sequence elements by DNA binding proteins associated with Polycomb repressive complex 2 (PRC2). However, the sequences that recruit PRC2 in mammalian cells have remained obscure. To address this, we integrated a series of engineered bacterial artificial chromosomes into embryonic stem (ES) cells and examined their chromatin. We found that a 44 kb region corresponding to the Zfpm2 locus initiates *de novo* recruitment of PRC2. We then pinpointed a CpG island within this locus as both necessary and sufficient for PRC2 recruitment. Based on this causal demonstration and prior genomic analyses, we hypothesized that large GC-rich elements depleted of activating transcription factor motifs mediate PRC2 recruitment in mammals. We validated this model in two ways. First, we showed that a constitutively active CpG island is able to recruit PRC2 after excision of a cluster of activating motifs. Second, we showed that two 1 kb sequence intervals from the *Escherichia coli* genome with GC-contents comparable to a mammalian CpG island are both capable of recruiting PRC2 when integrated into the ES cell genome. Our findings demonstrate a causal role for GC-rich sequences in PRC2 recruitment and implicate a specific subset of CpG islands depleted of activating motifs as instrumental for the initial localization of this key regulator in mammalian genomes.

## Author Summary

Key developmental genes are precisely turned on or off during development, thus creating a complex, multi-tissue embryo. The mechanism that keeps genes off, or repressed, is crucial to proper development. In embryonic stem cells, Polycomb repressive complex 2 (PRC2) is recruited to the promoters of these developmental genes and helps to maintain repression in the appropriate tissues through development. How PRC2 is initially recruited to these genes in the early embryo remains elusive. Here we experimentally demonstrate that stretches of GC-rich DNA, termed CpG islands, can initiate recruitment of PRC2 in embryonic stem cells when they are transcriptionally-inactive. Surprisingly, we find that GC-rich DNA from bacterial genomes can also initiate recruitment of PRC2 in embryonic stem cells. This supports a model where inactive GC-rich DNA can itself suffice to recruit PRC2 even in the absence of more complex DNA sequence motifs.

## Introduction

Polycomb proteins are epigenetic regulators required for proper gene expression patterning in metazoans. The proteins reside in two main complexes, termed Polycomb repressive complex 1 and 2 (PRC1 and PRC2). PRC2 catalyzes histone H3 lysine 27 tri-methylation (K27me3), while PRC1 catalyzes histone H2A ubiquitination and mediates chromatin compaction [1], [2]. PRC1 and PRC2 are initially recruited to target loci in the early embryo where they subsequently mediate lineage-specific gene repression. In embryonic stem (ES) cells, the complexes localize to thousands of genomic sites, including many developmental loci [3]–[5]. These target loci are not yet stably repressed, but instead maintain a “bivalent” chromatin state, with their chromatin enriched for the activating histone mark, H3 lysine 4 tri-methylation (K4me3), together with the repressive K27me3 [6], [7]. In the absence of transcriptional induction, PRC1 and PRC2 remain at target loci and mediate repression through differentiation. The mechanisms that underlie stable association of the complexes remain poorly understood, but likely involve interactions with the modified histones [8]–[12].

Proper localization of PRC1 and PRC2 in the pluripotent genome is central to the complex developmental regulation orchestrated by these factors. However, the sequence determinants that underlie this initial landscape remain obscure. Polycomb recruitment is best understood in *Drosophila*, where sequence elements termed Polycomb response elements (PREs) are able to direct these repressors to exogenous locations [13]. PREs contain clusters of motifs recognized by DNA binding proteins such as Pho, Zeste and GAGA, which in turn recruit PRC2 [14]–[17]. Despite extensive study, neither PRE sequence motifs nor binding profiles of PRC2-associated DNA binding proteins are sufficient to fully predict PRC2 localization in the *Drosophila* genome [1], [16], [18], [19].

While protein homologs of PRC1 and PRC2 are conserved in mammals, DNA sequence homologs of *Drosophila* PREs appear to be lacking in mammalian genomes [13]. Moreover, it remains controversial whether the DNA binding proteins associated with PRC2 in *Drosophila* have functional homologs in mammals. The most compelling candidate has been YY1, a Pho homolog that rescues gene silencing when introduced into Pho-deficient *Drosophila* embryos [20]. YY1 has been implicated in PRC2-dependent silencing of tumor suppressor genes in human cancer cells [21]. However, this transcription factor has also been linked to numerous other functions, including imprinting, DNA methylation, B-cell development and ribosomal protein gene transcription [22]–[26].

Recently, researchers identified two DNA sequence elements able to confer Polycomb repression in mammalian cells. Sing and colleagues identified a murine PRE-like element that regulates the MafB gene during neural development [27]. These investigators defined a critical 1.5 kb sequence element that is able to recruit PRC1, but not PRC2 in a transgenic cell assay. Woo and colleagues identified a 1.8 kb region of the human HoxD cluster that recruits both PRC1 and PRC2 and represses a reporter construct in mesenchymal tissues [28]. Both groups note that their respective PRE regions contain YY1 motifs. Mutation of the YY1 sites in the HoxD PRE resulted in loss of PRC1 binding and partial loss of repression, while comparatively, deletion of a separate highly conserved region from this element completely abrogated PRC1 and PRC2 binding as well as repression [28].

In addition to these locus-specific investigations, genomic studies have sought to define PRC2 targets and determinants in a systematic fashion. The Ezh2 and Suz12 subunits have been mapped in mouse and human ES cells by chromatin immunoprecipitation and microarrays (ChIP-chip) or high-throughput sequencing (ChIP-Seq) [3]–[5],[29]. Such studies have highlighted global correlations between PRC2 targets and CpG islands [5], [30] as well as highly-conserved genomic loci [4], [7], [31]. Recently, Jarid2 has been shown to associate with PRC2 and to be required for proper genome-wide localization of the complex [32]–[35]. Intriguingly, Jarid2 contains an ARID and a Zinc-finger DNA-binding domain. However, it is unclear how Jarid2 could account for PRC2 targeting given the lack of sequence specificity and the low affinity of its DNA binding domains [33], [36]. In summary, a variety of sequence elements including CpG islands, conserved elements and YY1 motifs have been implicated in Polycomb targeting in mammalian cells. Causality has only been demonstrated in two specific instances and a unifying view of the determinants of Polycomb recruitment remains elusive.

Here we present the identification of multiple sequence elements capable of recruiting PRC2 in mammalian ES cells. This was achieved through an experimental approach in which engineered bacterial artificial chromosomes (BACs) were stably integrated into the ES cell genome. Evaluation of a series of modified BACs specifically identified a 1.7 kb DNA fragment that is both necessary and sufficient for PRC2 recruitment. The fragment does not share sequence characteristics of *Drosophila* PREs and lacks YY1 binding sites, but rather corresponds to an annotated CpG island. Based on this result and a genome-wide analysis of PRC2 target sequences we hypothesized that large GC-rich sequence elements lacking transcriptional activation signals represent *general* PRC2 recruitment elements. We tested this model by assaying the following DNA sequences: (i) a ‘housekeeping’ CpG island which was re-engineered by removal of a cluster of activating motifs; and (ii) two large GC-rich intervals from the *E. coli* genome that satisfy the criteria of mammalian CpG islands. We found that all three GC-rich elements robustly recruit PRC2 in ES cells. We propose that a class of CpG islands distinguished by a lack of activating motifs play causal roles in the initial localization of PRC2 and the subsequent coordination of epigenetic controls during mammalian development.

## Results

### Recruitment of Polycomb repressors to a bacterial artificial chromosome integrated into ES cells

To identify DNA sequences capable of recruiting Polycomb repressors in mammalian cells, we engineered human BACs that correspond to genomic regions bound by these proteins in human ES cells.

**Figure 1:**
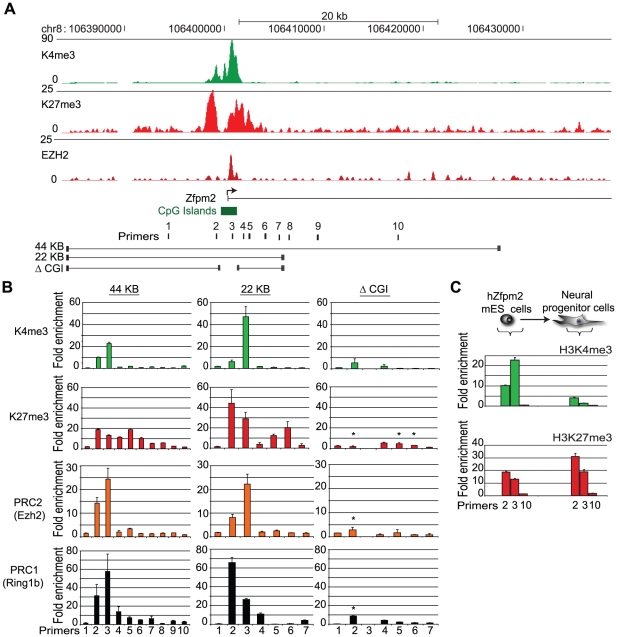
Recruitment of Polycomb repressors to a BAC integrated into ES cells. (A) ChIP-Seq tracks depict enrichment of K27me3 (the modification catalyzed by PRC2), Ezh2 (the enzymatic component of PRC2), and K4me3 across the endogenous hZfpm2 locus in human ES cells. Primers and constructs used in this study are indicated below the gene track. (B) BAC constructs from (A) containing the hZfpm2 locus were stably integrated into mouse ES cells. ChIP-qPCR enrichments are shown for K4me3, K27me3, Ezh2, and the PRC1 component Ring1b across the locus. The integrated locus adopts a ‘bivalent’ chromatin state with K27me3 and K4me3 in all constructs except the ΔCGI BAC. The locations of PCR amplicons are designated on the horizontal axis. (C) Transgenic ES cells differentiated along a neural lineage show enrichment for K27me3 but not K4me3 in NP cells. Error bars show standard error of the mean (SEM) for n = 3 (44 kb) or n = 2 (22 kb; ΔCGI) biological replicates.

We initially targeted a region of the human Zfpm2 (hZfpm2) locus, which encodes a developmental transcription factor involved in heart and gonad development [37]. In ES cells, the endogenous locus recruits PRC1 and PRC2, and is enriched for the bivalent histone modifications, K4me3 and K27me3 (Figure 1A). We used recombineering to engineer a 44 kb BAC containing this locus and a neomycin selection marker. The modified BAC was electroporated into mouse ES cells, and individual transgenic ES cell colonies containing the full length BAC were expanded (Figure S1). Fluorescent *in situ* hybridization (FISH) confirmed integration at a single genomic location (Figure S2).

We used ChIP and quantitative PCR (ChIP-qPCR) with human specific primers to examine the chromatin state of the newly incorporated hZfpm2 locus. This analysis revealed strong enrichment for K27me3 and K4me3 (Figure 1B). In addition, we explicitly tested for direct binding of the Polycomb repressive complexes using antibody against the PRC1 subunit, Ring1B, or the PRC2 subunit, Ezh2. We detected robust enrichment for both complexes in the vicinity of the hZfpm2 gene promoter (Figure 1B). To confirm this result and eliminate the possibility of integration site effects, we tested two additional transgenic hZfpm2 ES cell clones with unique integration sites as well as a fourth transgenic ES cell line containing a distinct Polycomb target locus, Pax5. In each case, we observed a bivalent chromatin state analogous to the endogenous loci (Figure S3). Similar to endogenous bivalent CpG islands, we found the Zfpm2 CpG island was DNA hypomethylated (Figure S4). These results suggest that DNA sequence is sufficient to initiate *de novo* recruitment of Polycomb in ES cells.

### The Zfpm2 BAC maintains K27me3 through ES cell differentiation

A key function of Polycomb repressors is to maintain a repressive chromatin state through cellular differentiation. To determine if the integrated BAC is capable of maintaining K27me3, the hZfpm2 transgenic ES cells were differentiated to neural progenitor (NP) cells *in vitro* [38]. ChIP-qPCR analysis revealed continued enrichment of K27me3 but loss of K4me3 (Figure 1C), a pattern frequently observed at endogenous loci that are not activated during differentiation [39].This indicates that DNA sequence at the hZfpm2 locus is sufficient to initiate K27me3 chromatin modifications in ES cells, and maintain the repressive chromatin state through neural differentiation.

### Distinguishing Polycomb recruiting sequences in the Zfpm2 BAC

We next sought to define the sequences within the hZfpm2 BAC required for recruitment of Polycomb repressors. First, we re-engineered the 44 kb hZfpm2 BAC to remove 20 kb of flanking sequences that contained distal non-coding conserved sequence elements (Figure 1A). When we integrated the resulting 22 kb construct into ES cells we found that it robustly enriches for PRC1, PRC2, K4me3 and K27me3 (Figure 1B). Hence, these particular distal elements do not appear to be required for the recruitment of the complexes. Next, we considered the necessity of the CpG island which corresponds to the peak of Ezh2 enrichment in ChIP-Seq profiles (Figure 1A). We excised a 1.7 kb fragment containing the CpG island, and integrated the resulting BAC (ΔCGI) into ES cells. The ΔCGI BAC failed to recruit PRC1 or PRC2, and showed significantly reduced K27me3 levels relative to the other constructs (Figure 1B). This suggests that the CpG island is essential for recruitment of Polycomb proteins to the hZfpm2 locus.

### A 1.7 kb CpG island is sufficient to recruit PRC2 to an exogenous locus

**Figure 2:**
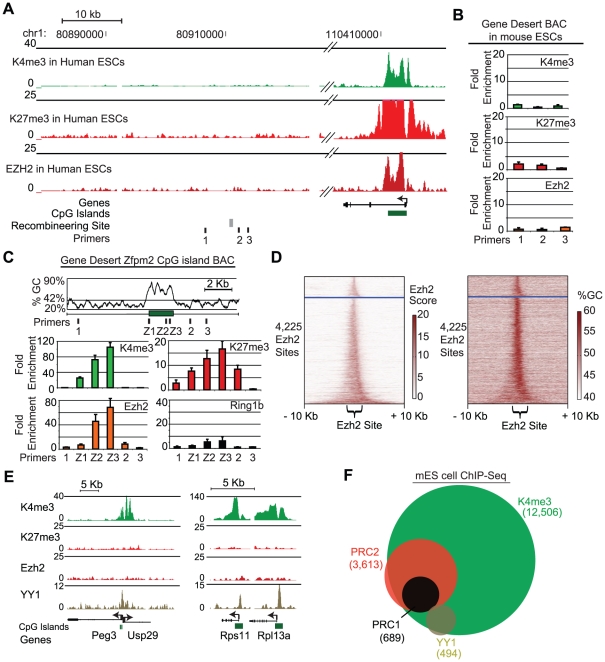
A 1.7 kb GC-rich sequence element is sufficient to recruit PRC2. (A) ChIP-Seq tracks show no enrichment for K4me3, K27me3 or Ezh2 in human ES cells across the gene desert region. For comparison a nearby locus is shown. The recombineering site and primers used in this study are indicated below the tracks. (B) The gene desert BAC shows no enrichment of K4me3, K27me3 or PRC2 upon integration in mouse ES cells. (C) The hZfpm2 CpG island is depicted at the site of insertion into the gene desert BAC, along with the corresponding GC percentage (42% indicates genome average) and primers used for qPCR. Underlying plots represent ChIP-qPCR enrichment of K4me3, K27me3, PRC2 (Ezh2), and PRC1 (Ring1b) at the indicated sites (n = 2 biological replicates). (D) Heat maps show Ezh2 ChIP-Seq signal (left panel) or GC-percentage (right panel) for all Ezh2-bound regions in ES cells. Each row depicts a 20 kb region centered on the Ezh2 signal. Rows are separated into two groups based on whether the site overlaps a CpG island (below the blue line) and are then sorted based on the width of Ezh2 enrichment (see Methods). (E) ChIP-Seq was used to profile the mammalian Pho homolog YY1 in mouse ES cells. Genome browser views show ChIP-Seq enrichment signals for K4me3, K27me3, Ezh2 and YY1 for YY1 target loci. (F) Venn diagram shows overlap of K4me3, Ezh2, Ring1b, and YY1 at promoters in mES cells.

We next asked whether the hZfpm2 CpG island is sufficient to recruit Polycomb repressors to an exogenous locus. To test this, we selected an unremarkable gene desert region on human chromosome 1 that shows no enrichment for PRC1, PRC2 or K27me3 in ES cells (Figure 2A). We also verified that the gene desert BAC alone does not show any enrichment for K27me3 or Ezh2 when integrated into ES cells (Figure 2B). Using recombineering, we inserted the 1.7 kb sequence that corresponds to the hZfpm2 CpG island into the gene desert BAC. The resulting construct was integrated into mouse ES cells and three independent clones were evaluated. ChIP-qPCR analysis revealed strong enrichment for K27me3, K4me3 and PRC2 over the inserted CpG island (Figure 2C, Figure S5). In contrast, we observed relatively little enrichment for the PRC1 subunit Ring1B (Figure 2C). We confirmed the specificity of these enrichments with primers that span the boundary between the insertion and adjacent gene desert sequence. Notably, K27me3 enrichment was detected across the gene desert locus up to 2.5 kb from the inserted CpG island (Figure 2C). This indicates that the localized CpG island can initiate K27me3 that then spreads into adjacent sequence. Lastly we found no YY1 enrichment across the CpG island by ChIP-qPCR (Figure S5). Together, these data suggest that the hZfpm2 CpG island contains the necessary signals for PRC2 recruitment but is insufficient to confer robust PRC1 association.

### Consideration of sequence determinants of PRC2 recruitment

The functionality of a CpG island in PRC2 recruitment is consistent with prior observations that a majority of PRC2 sites in ES cells correspond to CpG islands [4], [5] and with the striking correlation between intensity of PRC2 binding and the GC-richness of the underlying sequence (Figure 2D). We therefore considered whether specific signals within the Zfpm2 CpG island might underlie its capacity to recruit PRC2.

First, we searched for sequence motifs analogous to the PREs that recruit PRC2 in *Drosophila*. We focused on motifs recognized by YY1, the nearest mammalian homolog of the *Drosophila* recruitment proteins. Notably, both of the recently described mammalian PREs contain YY1 motifs [27], [28]. The 44 kb hZfpm2 BAC contains 11 instances of the consensus YY1 motif. However, none of these reside within the CpG island (Figure S6) (see Methods). We also examined YY1 binding directly in ES cells and NS cells using ChIP-Seq. Consistent with prior reports, YY1 binding is evident at the 5′ ends of many highly expressed genes, including those encoding ribosomal proteins, and is also seen at the imprinted Peg3 locus (Figure 2E, Table S1) [26]. However, no YY1 enrichment is evident at the Zfpm2 locus. Moreover, at a global level, YY1 shows almost no overlap with PRC2 or PRC1, but instead co-localizes with genomic sites marked exclusively by K4me3 (Figure 2F, Figure S6, and Table S1). Thus, although YY1 may contribute to Polycomb-mediated repression through distal interactions or in *trans*, it does not appear to be directly involved in PRC2 recruitment in ES cells.

We previously reported that CpG islands bound by PRC2 in ES cells could be predicted based on a relative absence of activating transcription factor motifs (AMs) in their DNA sequence [5]. We reasoned that transcriptional inactivity afforded by this absence of AMs is a requisite for PRC2 association [40], [41]. This could explain why PRC2 is absent from a majority of CpG islands, many of which are found at highly active promoters. Consistent with this model, when we examined a recently published RNA-Seq dataset for poly-adenylated transcripts in ES cells, we found that virtually all of the high-CpG promoters (HCPs) lacking Ezh2 are detectably transcribed (Figure S7). The small proportion of HCPs that are neither Ezh2-bound nor transcribed may reflect false-negatives in the ChIP-Seq or RNA-Seq data. Alternatively, these HCPs tend to correspond to CpG islands with relatively low GC-contents and lengths and may therefore have insufficient GC-richness to promote PRC2 binding (Figure S7). Thus, correlative analyses implicate large GC-rich elements that lack transcriptional activation signals as general PRC2 recruitment elements in mammals.

### Sufficiency of GC-rich sequences for PRC2 recruitment

**Figure 3:**
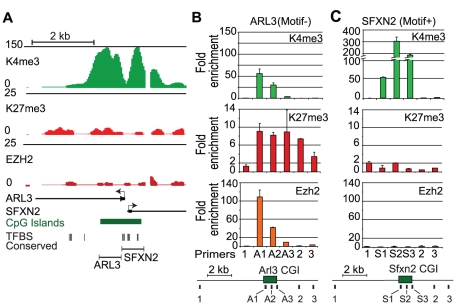
Removal of activating transcription factor motifs initiates PRC2 recruitment. (A) Genome browser views shows a locus containing the promoters for the housekeeping genes Arl3 and Sfxn2 with ChIP-Seq enrichment signals for K4me3, K27me3, and Ezh2 in mouse ES cells. This region contains a 1.8 kb CpG island that has the transcription factor motifs clustered on one side. Below shows the regions used for integration into the gene desert BAC. (B) After integration into mouse ES cells, ChIP-qPCR was conducted using three primers from the CpG island inserts and 3 primers in the flanking gene desert sequence. The motif devoid Arl3 section shows *de novo* PRC2 (Ezh2) recruitment and K4me3 and K27me3 enrichment. (C) The motif containing Sfxn2 half shows no enrichment for K27me3 but significant enrichment for K4me3, similar to the endogenous locus shown in (A) (n = 2 biological replicates).

To obtain direct experimental support for the general sufficiency of large GC-rich elements lacking AMs in PRC2 recruitment, we carried out the following experiments. First, we tested whether a K4me3-only CpG island could be turned into a PRC2 recruitment element by removing activating motifs. We targeted a 1.3 kb CpG island that overlaps the promoters of two ubiquitously expressed genes – Arl3 and Sfxn2. Neither gene carries K27me3 in ES cells, or in any other cell type tested (Figure S8, and data not shown). This CpG island was selected as it has many conserved AMs clustered in one half of the island (Figure 3A). We hypothesized that the portion of the Arl3/Sfxn2 CpG island lacking AMs would, in isolation, lack active transcription and recruit PRC2. In contrast, we predicted that the half containing multiple AMs would lack Polycomb. To test this, we generated two additional BAC constructs containing the respective portions of the Arl3/Sfxn2 CpG island positioned within the gene desert, and integrated these constructs into ES cells (Figure 3A). ChIP-qPCR shows that the portion of the CpG island lacking AMs is able to recruit PRC2 and becomes enriched for K27me3 (Figure 3B). In contrast, the AM-containing portion shows no enrichment for K27me3 or Ezh2, but is instead marked exclusively by K4me3, similar to the endogenous human locus (Figure 3C, Figure S8). Thus, a GC-rich sequence element with no known requirement for Polycomb regulation can recruit PRC2 when isolated from activating sequence features.

**Figure 4:**
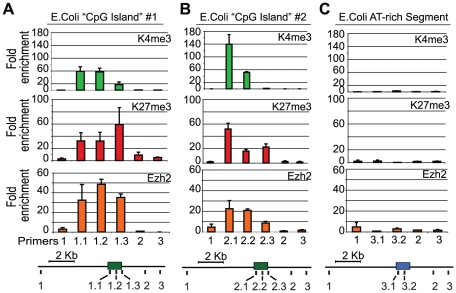
PRC2 is recruited to E.coli GC-rich sequences in mouse ES cells. The E. coli genome was scanned for 1 kb regions that met the criteria for a mammalian CpG island and had few motifs for mammalian transcription factors (see Methods). (A,B) Both GC-rich segments adopt a ‘bivalent’ chromatin state with K27me3 and K4me3 and recruit PRC2 (Ezh2) upon integration in mouse ES cells. (C) A non-CG rich region of the E. coli genome failed to recruit Ezh2 and lacked K4me3 and K27me3 (n = 2 biological replicates).

Next, we tested whether even more generic GC-rich elements might also be capable of recruiting PRC2 in ES cells. Here, we focused on sequences derived from the genome of *E. coli*, reasoning that there would be no selection for PRC2 recruiting elements in this prokaryote given the complete lack of chromatin regulators. We arbitrarily selected three 1 kb segments of the *E. coli* genome. Two with GC contents above the threshold for a mammalian CpG island but that each contained few AMs, and one AT rich segment as a control (Table S3). We recombined each segment into the gene desert BAC and integrated the resulting constructs into ES cells. ChIP-qPCR confirmed that both GC-rich *E. Coli* segments recruit Ezh2 and form a bivalent chromatin state (Figure 4A, 4B, Figure S9). Notably, the GC-rich segment also enriches for Jarid2, a PRC2 component with DNA binding activity (Figure S10). In contrast, the AT-rich segment did not recruit Ezh2 or enrich for either K4me3 or K27me3 (Figure 4C, Figure S9). Together, our findings suggest that GC-rich sequence elements that lack signals for transcriptional activation have an innate capacity to recruit PRC2 in mammalian ES cells.

## Discussion

Several lines of evidence suggest that the initial landscape of Polycomb complex binding is critical for proper patterning of gene expression in metazoan development [1], [2], [13]. Failure of these factors to engage their target loci in embryogenesis has been linked to a loss of epigenetic repression at later stages. Accordingly, the determinants that localize Polycomb complexes at the pluripotent stage are almost certainly essential to the global functions of these repressors through development.

We find that DNA sequence is sufficient for proper localization of Polycomb repressive complexes in ES cells, and specifically identify a CpG island within the Zfpm2 locus as being critical for recruitment. We provide evidence that GC-rich elements lacking activating signals suffice *in general* to recruit PRC2. This includes demonstrations (i) that a motif devoid segment of an active ‘housekeeping’ CpG island can recruit PRC2; and (ii) that arbitrarily selected GC-rich elements from the *E. coli* genome can themselves mediate PRC2 recruitment when integrated into the ES cell genome.

**Figure 5:**
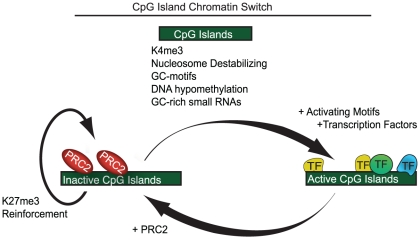
A model showing CpG islands as a chromatin switch. (A) Features common to both active and inactive CpG islands include destabalization of nucleosomes, simple GC-motifs, K4me3 and lack of DNA methylation. Additionally, many CpG island transcribe small non-coding GC-rich RNAs. Active CpG islands contain motifs associated with numerous activating transcription factors and transcriptional machinery, which likely prevent PRC2 from binding. In contrast, CpG islands lacking activating motifs are bound by PRC2 which, through a positive feedback loop with K27me3, maintains an inactive state.

Several possible mechanistic models could explain the causality of GC-rich DNA elements in PRC2 recruitment (Figure 5). First, we note that CpG islands have been shown to destabilize nucleosomes in mammalian cells [42]. At transcriptionally inactive loci, this property could increase their accessibility to PRC2-associated proteins with DNA affinity but low sequence specificity, such as Jarid2 or AEBP2 [32]–[35], [43] (Figure S10). Although this association would be abrogated by transcriptional activity at most CpG islands, those lacking activation signals would remain permissive to PRC2 association (Figure 5). In support of this model, PRC2 targets in ES cells are also enriched for H2A.Z and H3.3, histone variants linked to nucleosome exchange dynamics [44], [45]. Alternatively or in addition, targeting could be supported by DNA binding proteins with affinity for low complexity GC-rich motifs or CpG dinucleotides, such as CXXC domain proteins [46]. Localization may also be promoted or stabilized by long and short non-coding RNAs [47]–[50] as well as by the demonstrated affinity of PRC2 for its product, H3K27me3 [11], [12]. Notably, PRC2 recruitment in ES cells appears distinct from that in *Drosophila*, as we do not find evidence for involvement of PRE-like sequence motifs or mammalian homologues such as YY1.

It should be emphasized that PRC2 localization does not necessarily equate with epigenetic repression. Indeed virtually all PRC2 bound sites in ES cells, and all CpG islands tested here, are also enriched for K4me3, and presumably poised for activation upon differentiation. Epigenetic repression during differentiation may require PRC1 and thus depend on additional binding determinants. YY1 remains an intriguing candidate in this regard, given prior evidence for physical and genetic interactions with PRC1 [51], [52]. YY1 consensus motifs are present in the Polycomb-dependent silencing elements recently identified in the MafB and HoxD loci. Interestingly, the HoxD element combines a CpG island with a cluster of conserved YY1 motifs. Mutation of the motifs abrogated PRC1 binding but left PRC2 binding intact. Still, the fact that only a small fraction of documented PRC2 and PRC1 sites have YY1 motifs or binding suggests that this transcription factor may act indirectly and/or explain only a subset of cases. Nonetheless, it is likely that a fully functional epigenetic silencer would require a combination of features, including a GC-rich PRC2 element as well as appropriate elements to recruit PRC1. Further study is needed to expand the rules for PRC2 binding to include a global definition of PRC1 determinants and ultimately, to understand how the initial landscape facilitates the maintenance of gene expression programs in the developing organism.

## Methods

### BAC construct design

BAC constructs CTD331719L (‘Zfpm2 44’), CTD-2535J16 (‘Pax5’) and CTD-3219L19 (‘Gene Desert’) were obtained from Open Biosystems. Recombineering was done using the RedET system (Open Biosystems) in DH10B cells. Homology arms 200–500 bp in length were PCR amplified and cloned into a PGK; Neomycin cassette (Gene Bridges). This cassette was used to recombineer all BACs to enable selection in mammalian cells. The 22 kb hZfpm2 BAC was created by restricting the hZfpm2 BAC at two sites using ClaI, and re-ligating the BAC lacking the intervening sequence. The CpG island was excised from the 22 kb hZfpm2 BAC by amplification of flanking homology arms, and cloned into a construct containing an adjacent ampicillin cassette (Frt-amp-Frt; Gene Bridges). After recombination, the ampicillin cassette was removed using Flp-recombinase and selection for clones that lost ampicillin resistance (Flp-706; Gene Bridges). PCR across the region confirmed excision of the CpG island. For the Gene Desert BACs, the Zfpm2, Arl3, Sfxn2 and E. coli CpG islands were amplified with primers containing XhoI sites and cloned into the Frt-amp-Frt vector that contains homology arms from the Gene Desert region. The final constructs were confirmed by sequencing across recombination junctions. All primers used for CpG islands and recombineering homology arms are listed in Table S2.

### Transgenic ES cell and ChIP experiments

ES cells (V6.5) were maintained in ES cell medium (DMEM; Dulbecco's modified Eagle's medium) supplemented with 15% fetal calf serum (Hyclone), 0.1 mM ß-mercaptoethanol (Sigma), 2 mM Glutamax, 0.1 mM non-essential amino acid (NEAA; Gibco) and 1000U/ml recombinant leukemia inhibitory factor (ESGRO; Chemicon). Roughly 50 µg of linearized BAC was nucleofected using the mouse ES cell nucleofector kit (Lonza) into 10^6^ mouse ES cells, and selected 7–10 days with 150 µg/ml Geneticin (Invitrogen) on Neomycin resistant MEFs (Millipore). Individual resistant colonies were picked, expanded and tested for integration of the full length BAC by PCR. Differentiation of hZfpm2 ES cell clone 1 into a population of neural progenitor (NP) cells was done as previously described [53]. FISH analysis was done as described previously [54]. DNA methylation analysis was done as previously described [55] and primers used to amplify bisulfite treated DNA are listed in Table S2.

For each construct, between one and three ES cell clones were expanded and subjected to ChIP using antibody against K4me3 (Abcam ab8580 or Upstate/Millipore 07-473), K27me3 (Upstate/Millipore 07-449), Ezh2 (Active Motif 39103 or 39639), or Ring1B (MBL International d139-3) as described previously [5], [7], [39]. ChIP DNA was quantified by Quant-iT Picogreen dsDNA Assay Kit (Invitrogen). ChIP enrichments were assessed by quantitative PCR analysis on an ABI 7500 with 0.25 ng ChIP DNA and an equal mass of un-enriched input DNA. Enrichments were calculated from 2 or 3 biologically independent ChIP experiments. For K27me3, and Ezh2 enrichment, background was subtracted by normalizing over a negative genomic control. Error bars represent standard error of the mean (SEM). We confirmed that the human specific primers do not non-specifically amplify mouse genomic DNA. Primers used for qPCR are listed in Table S2.

### Genomic and computational analysis

Genomewide maps of YY1 binding sites were determined by ChIP-Seq as described previously [39]. Briefly, ChIP was carried out on 6×10^7^ cells using antibody against YY1 (Santa Cruz Biotechnology sc-1703). ChIP DNA was used to prepare libraries which were sequenced on the Illumina Genome Analyzer. Density profiles were generated as described [39]. Promoters (RefSeq; http://genome.ucsc.edu) were classified as positive for YY1, H3K4me3 or H3K27me3 if the read density was significantly enriched (p<10^−3^) over a background distribution based on randomized reads generated separately for each dataset to account for the varying degrees of sequencing depth. ChIP-Seq data for YY1 are deposited to the NCBI GEO database under the following accession number GSE25197 (http://www.ncbi.nlm.nih.gov/projects/geo/query/acc.cgi?acc=GSE25197). Sites of Ezh2 enrichment (p<10^−3^) were calculated genomewide using sliding 1 kb windows, and enriched windows within 1 kb were merged. DNA methylation levels were calculated using previously published Reduced Representation Bisulphite Sequenced (RRBS) libraries [55]. Composite plots represent the mean methylation level in sliding 200 bp windows in the the 10 kb surrounding the TSSs of the indicated gene sets.

YY1 motifs were identified using the MAST algorithm [56] where a match to the consensus motif was defined at significance level 5×10^−5^. Candidate CpG islands for TF motif analysis were identified by scanning annotated CpG islands (http://genome.ucsc.edu) for asymmetric clustering of motifs related to transcriptional activation in ES cells [5]. Motifs shown in Figure 3A and Figure S6 are from UCSCs TFBS conserved track. GC-rich elements from the *E. coli* K12 genome were selected by calculating %GC and CpG O/E in sliding 1 kb windows. Sequences matching the criteria for mammalian CpG islands while simultaneously being depleted of motifs related to transcriptional activation [5] were chosen for insertion into mouse ES cells. Transcriptionally inactive HCPs were selected based on a lack of transcript enrichment by both expression arrays [39] and RNA-Seq data [57]. In the case of RNA-Seq, each gene was assigned the maximum read density within any 1 kb window of exonic sequence. To ease analysis of promoter CpG island statistics, only HCPs containing a single CpG island were considered.

## Supporting Information

Figure S1 A schematic of the transgenic chromatin assay that was used to examine the role of DNA sequence in determining histone modification patterns in embryonic stem cells.
(0.34 MB PDF)

Figure S2 Transgenic mouse ES cells and associated mouse feeder cells were probed by FISH using Human BAC CTD331719L (hZFPM2), labeled with Cy3-dUTP (red), and a control mouse probe BAC (RP23-442F1, located on mouse chromosome 15), labeled with FITC-dUTP (green) along with DNA stained with DAPI (blue). A MEF feeder cell (A) shows two copies of the mouse probe (green arrows), and lacks a copy of hZfpm2. A transgenic ES cell (B) shows two copies of the mouse probe (green arrows) and one copy of hZFPM2 probe (red arrow).
(0.51 MB PDF)

Figure S3 (A) Two additional mES cell clones containing the 44 kb hZfpm2 locus were examined using ChIP-qPCR similar to Figure 1C. Both show enrichment of H3K4me3 and H3K27me3 across the gene promoter. (B) ChIP-seq map of the human Pax5 locus in human ES cells show broad regions of H3K4me3 and H3K27me3 enrichment. Bottom panel shows ChIP-qPCR of transgenic mouse ES cells carrying a 50 kb region of the hPax5 locus showing a similar enrichment of H3K4me3 and H3K27me3 across the region. (Error bars represent SEM, n = 3). Primer numbers correspond to primer names in Table S2.
(0.25 MB PDF)

Figure S4 (A) Composite plots showing the lack of DNA methylation at both bivalent and K4me3 only promoters in mouse ES cells. (B) Schematic showing the CpG island of the Zfpm2 BAC remains free of DNA methylation upon integration into mouse ES cells. (C) The raw data used to create (B) shows aligned sequencing reads of Zfpm2 ES cell genomic DNA that was bisulfite treated (see Methods). Unmethylated and *in vitro* methylated BAC DNA are shown as controls. The underlined bases indicate sites of CG dinucletides.
(0.22 MB PDF)

Figure S5 (A) One additional mES cell clone containing 22 kb of the hZfpm2 locus was examined using ChIP-qPCR. As seen with the first clone (Figure 1B) this clone also shows enrichment of H3K4me3 and H3K27me3 at the gene promoter. (B) Additional clones of transgenic ES cells containing the Gene Desert BAC with the hZfpm2 CpG island inserted show enrichment of H3K4me3 and H3K27me3 as seen with clone #1 (Figure 2C). (C) The Zfpm2 Gene Desert BAC shows no enrichment of YY1, in contrast to the promoter of Rpl13a. Error bars equal to SEM (n = 2) Primer Key (see Table S3 for sequences): Genomic Ctrl = mouse neg genomic control
(0.15 MB PDF)

Figure S6 (A) The GC-richness and locations of YY1 motifs for the Zfpm2 locus are shown. (B) The 1.7 kb CpG island contains 4 conserved motifs (see Methods). (C) PRC2 signal is inversely correlated with YY1 signal at 17,761 promoters in mouse ES cells. (D) PRC2 activity as measured by K27me3 also shows an inverse correlation with YY1 in mouse neural stem (NS) cells. (E) Genome-wide binding profiles show YY1 is predominantly over 1 mb away from the nearest Ezh2 site. By comparison CpG islands (F) show close proximity to Ezh2 sites in ES cells.
(2.21 MB PDF)

Figure S7 (A) Analysis of gene promoters with high CpG content (HCPs) shows Ezh2 positive promoters have significantly lower RNA-Seq scores compared to Ezh2 negative promoters. The dashed line represents the highest expression seen at LCPs. All transcriptionally inactive HCPs containing a single CpG island were scored for Ezh2 enrichment (see text and Methods). (B) The scatter plot indicates length and %GC for Ezh2-positive and Ezh2-negative CpG islands with low RNA-Seq scores in mouse ES cells.
(0.41 MB PDF)

Figure S8 One additional mES cell clone containing gene desert BAC with the Sfxn2 CpG island was examined using ChIP-qPCR. As seen with the first clone (Figure 3B) this clone also shows significant enrichment of H3K4me3 but not H3K27me3 at the CpG island. Error Bars represent SEM (n = 2).
(0.15 MB PDF)

Figure S9 (A) One additional mES cell clone for each E. coli DNA construct was analyzed by ChIP-qPCR. As seen with the first clones (Figure 4A–4C) the CpG island clones show significant enrichment of K4me3, K27me3 and Ezh2 at the gene promoter. Error Bars represent SEM (n = 2) (B) As a negative control, E. Coli CpG island #1 was also tested for the chromatin modifiers Jarid1a and Kmt4, which showed no enrichment.
(0.22 MB PDF)

Figure S10 ChIP-qPCR shows Jarid2 enrichment signal at the CpG island (primer 6) of the 44 kb BAC (A), the Zfpm2 CpG island (primer Z1) within the Gene Desert BAC (B) and the GC-rich element (primers 1.1, 1.2) from E. Coli (C). Error Bars represent SEM (n = 2).
(0.15 MB PDF)

Table S1 YY1 bound sites in mouse ES cells and NS cells.
(0.07 MB XLS)

Table S2 Primer sequences used for recombineering and qPCR.
(0.02 MB XLS)

Table S3 Motifs and sequences of *E. Coli* GC-rich and AT control segments.
(0.01 MB XLS)
