# Supplementary material for: GC-Rich Sequence Elements Recruit PRC2 in Mammalian ES Cells
Source: PLoS Genet. 2010 Dec 9;6(12):e1001244. doi: 10.1371/journal.pgen.1001244 (PMC3000368; doi:10.1371/journal.pgen.1001244)
Supplement: Figure S1 — A schematic of the transgenic chromatin assay that was used to examine the role of DNA sequence in determining histone modification patterns in embryonic stem cells. (0.34 MB PDF) [file pgen.1001244.s001.pdf]

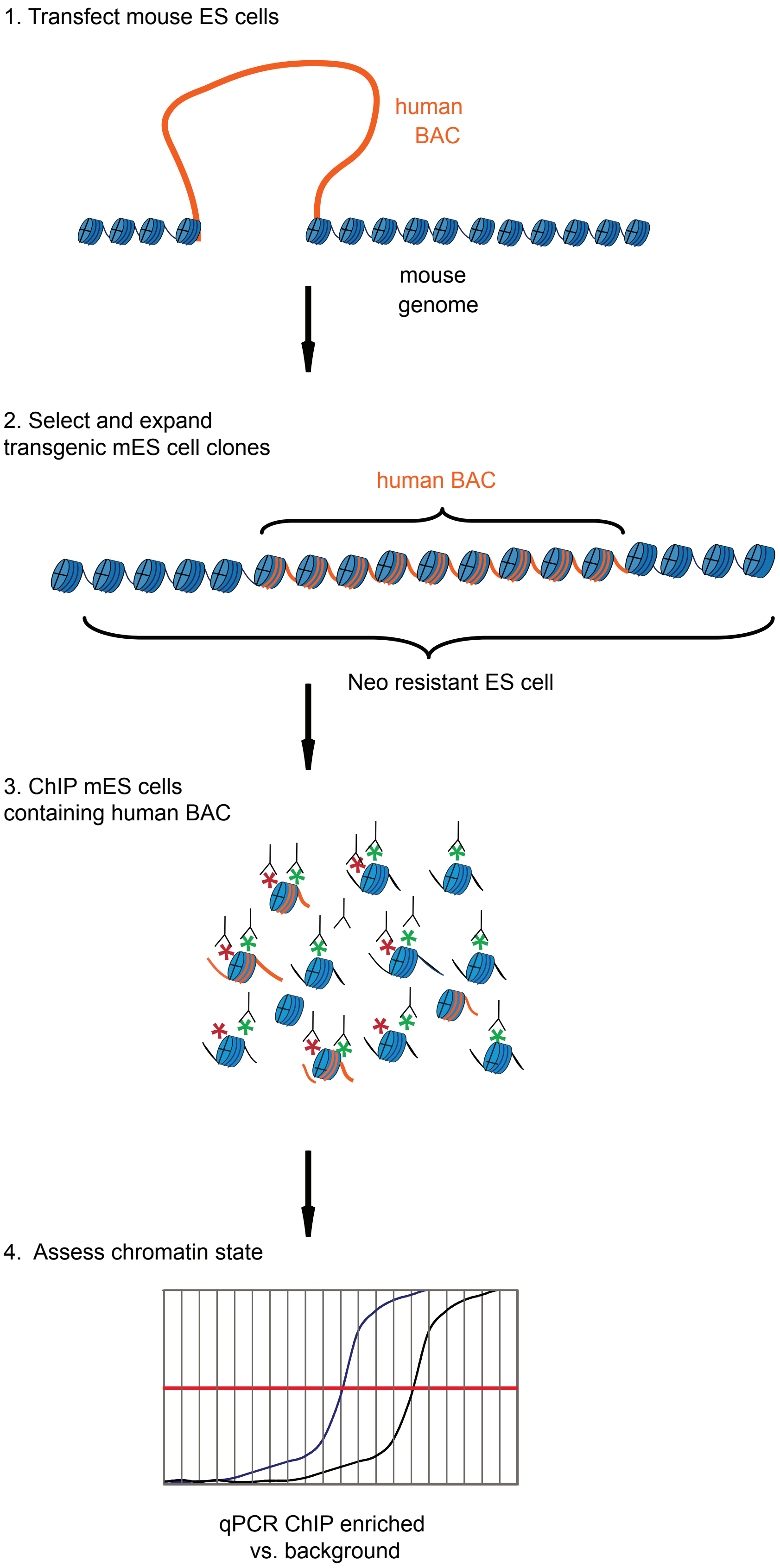
1. Transfect mouse ES cells
human
BAC
mouse
genome
2. Select and expand
transgenic mES cell clones
human BAC
Neo resistant ES cell
3. ChIP mES cells
containing human BAC
4. Assess chromatin state
qPCR ChIP enriched
vs. background
