# Supplementary figures and images for: GC-Rich Sequence Elements Recruit PRC2 in Mammalian ES Cells
Source: PLoS Genet. 2010 Dec 9;6(12):e1001244. doi: 10.1371/journal.pgen.1001244 (PMC3000368; doi:10.1371/journal.pgen.1001244)

**A**

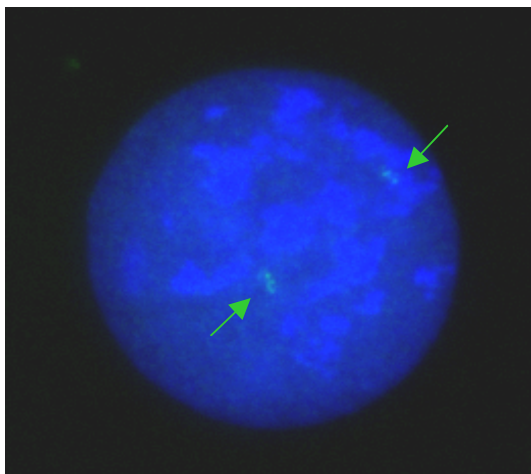

**B**

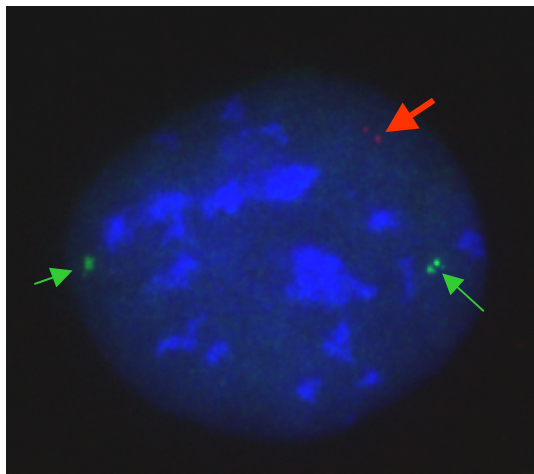

Supplement: Figure S2 — Transgenic mouse ES cells and associated mouse feeder cells were probed by FISH using Human BAC CTD331719L (hZFPM2), labeled with Cy3-dUTP (red), and a control mouse probe BAC (RP23-442F1, located on mouse chromosome 15), labeled with FITC-dUTP (green) along with DNA stained with DAPI (blue). A MEF feeder cell (A) shows two copies of the mouse probe (green arrows), and lacks a copy of hZfpm2. A transgenic ES cell (B) shows two copies of the mouse probe (green arrows) and one copy of hZFPM2 probe (red arrow). (0.51 MB PDF) [file pgen.1001244.s002.pdf]

**A**

hZfpm2 44 Kb ES Cell Clone #2

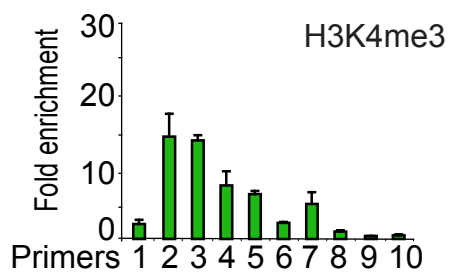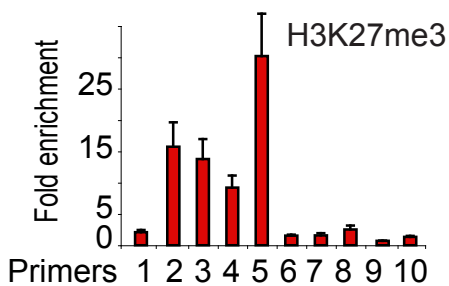

hZfpm2 44 Kb ES Cell Clone #3

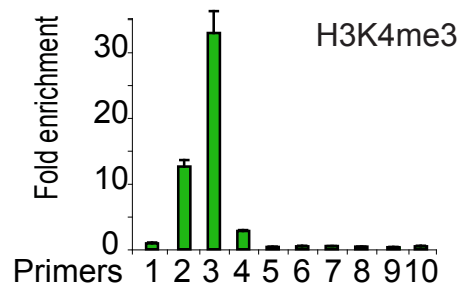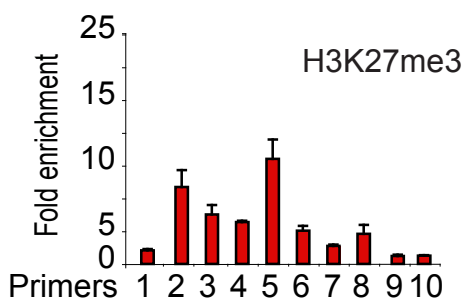**B**

hPax5 ES Cell Clone #1

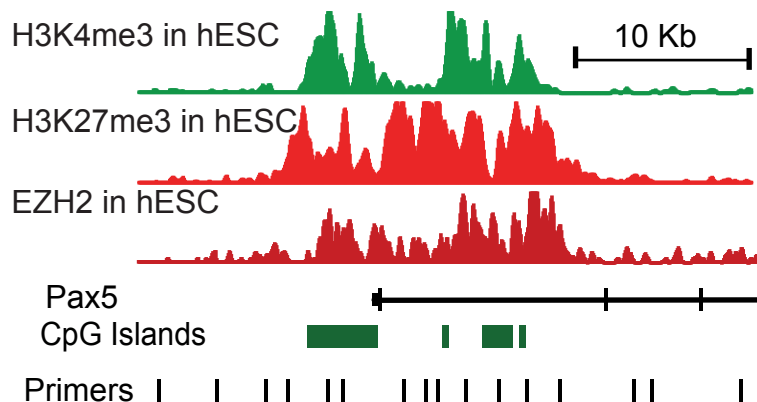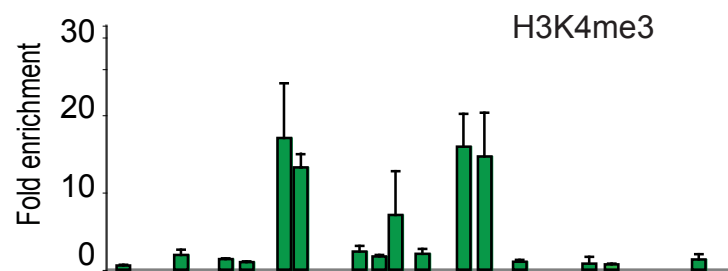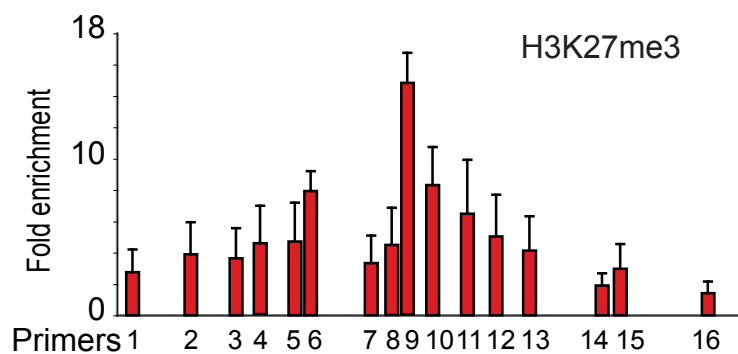

Supplement: Figure S3 — (A) Two additional mES cell clones containing the 44 kb hZfpm2 locus were examined using ChIP-qPCR similar to Figure 1C. Both show enrichment of H3K4me3 and H3K27me3 across the gene promoter. (B) ChIP-seq map of the human Pax5 locus in human ES cells show broad regions of H3K4me3 and H3K27me3 enrichment. Bottom panel shows ChIP-qPCR of transgenic mouse ES cells carrying a 50 kb region of the hPax5 locus showing a similar enrichment of H3K4me3 and H3K27me3 across the region. (Error bars represent SEM, n = 3). Primer numbers correspond to primer names in Table S2. (0.25 MB PDF) [file pgen.1001244.s003.pdf]

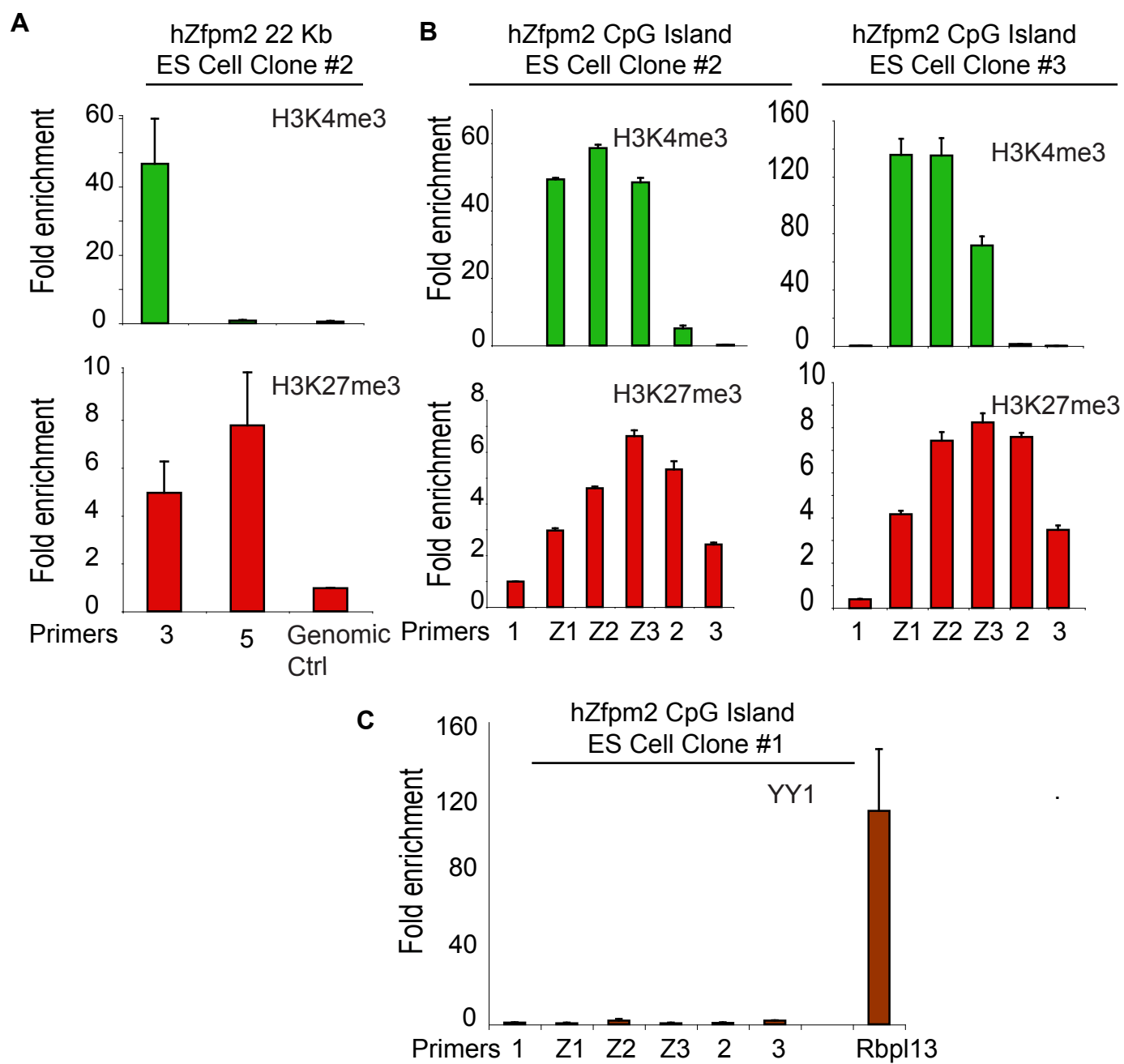

Supplement: Figure S5 — (A) One additional mES cell clone containing 22 kb of the hZfpm2 locus was examined using ChIP-qPCR. As seen with the first clone (Figure 1B) this clone also shows enrichment of H3K4me3 and H3K27me3 at the gene promoter. (B) Additional clones of transgenic ES cells containing the Gene Desert BAC with the hZfpm2 CpG island inserted show enrichment of H3K4me3 and H3K27me3 as seen with clone #1 (Figure 2C). (C) The Zfpm2 Gene Desert BAC shows no enrichment of YY1, in contrast to the promoter of Rpl13a. Error bars equal to SEM (n = 2) Primer Key (see Table S3 for sequences): Genomic Ctrl = mouse neg genomic control (0.15 MB PDF) [file pgen.1001244.s005.pdf]

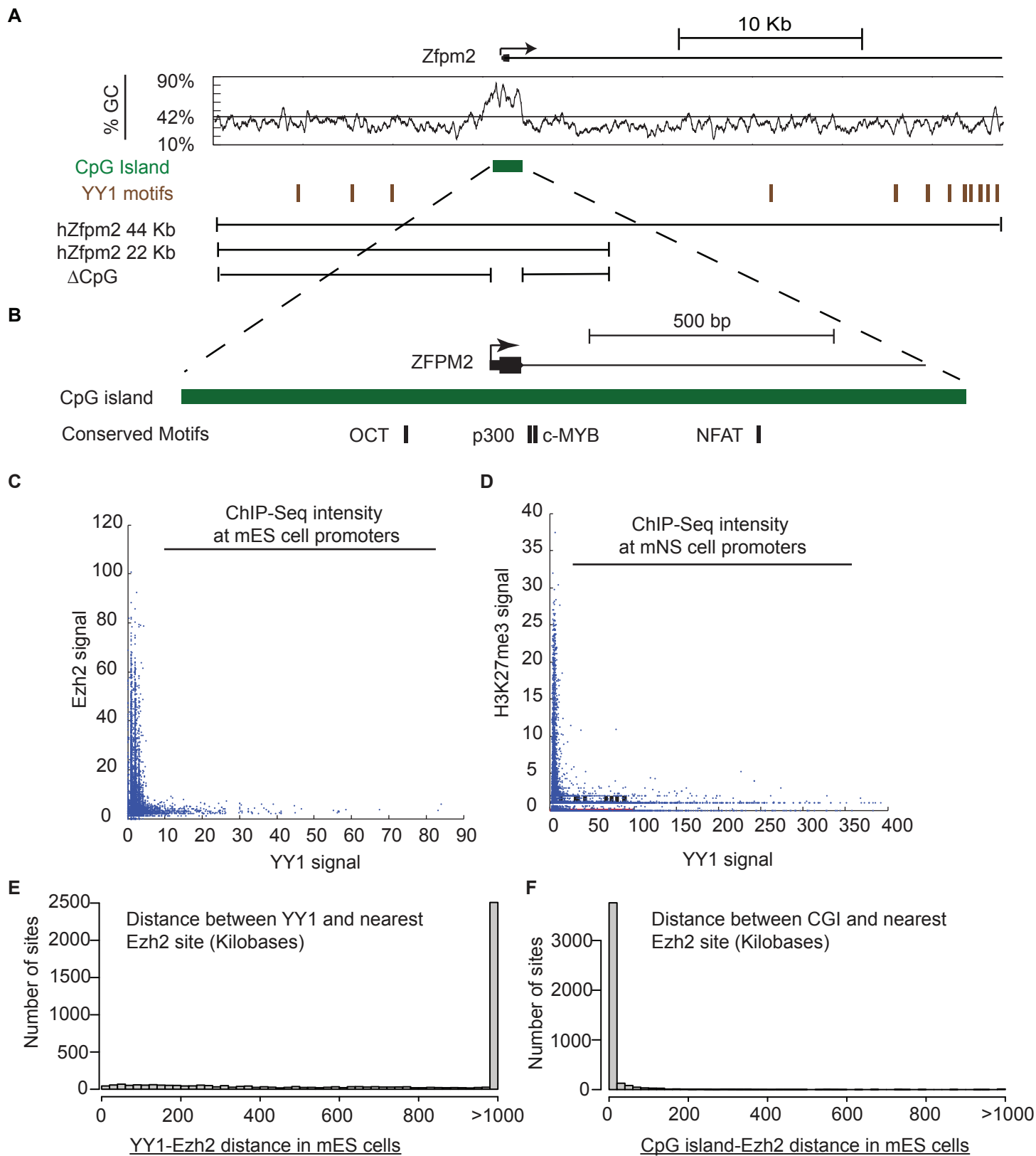

Supplement: Figure S6 — (A) The GC-richness and locations of YY1 motifs for the Zfpm2 locus are shown. (B) The 1.7 kb CpG island contains 4 conserved motifs (see Methods). (C) PRC2 signal is inversely correlated with YY1 signal at 17,761 promoters in mouse ES cells. (D) PRC2 activity as measured by K27me3 also shows an inverse correlation with YY1 in mouse neural stem (NS) cells. (E) Genome-wide binding profiles show YY1 is predominantly over 1 mb away from the nearest Ezh2 site. By comparison CpG islands (F) show close proximity to Ezh2 sites in ES cells. (2.21 MB PDF) [file pgen.1001244.s006.pdf]

**A**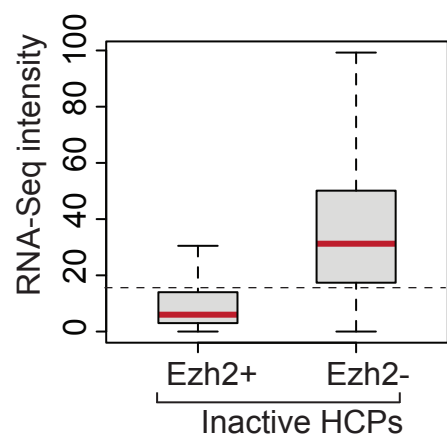**B**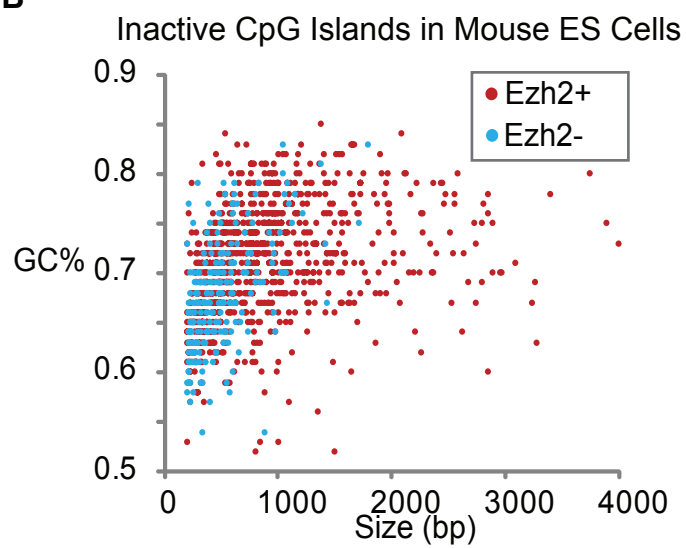

Supplement: Figure S7 — (A) Analysis of gene promoters with high CpG content (HCPs) shows Ezh2 positive promoters have significantly lower RNA-Seq scores compared to Ezh2 negative promoters. The dashed line represents the highest expression seen at LCPs. All transcriptionally inactive HCPs containing a single CpG island were scored for Ezh2 enrichment (see text and Methods). (B) The scatter plot indicates length and %GC for Ezh2-positive and Ezh2-negative CpG islands with low RNA-Seq scores in mouse ES cells. (0.41 MB PDF) [file pgen.1001244.s007.pdf]

### Gene Desert with Sfxn2 Clone #2

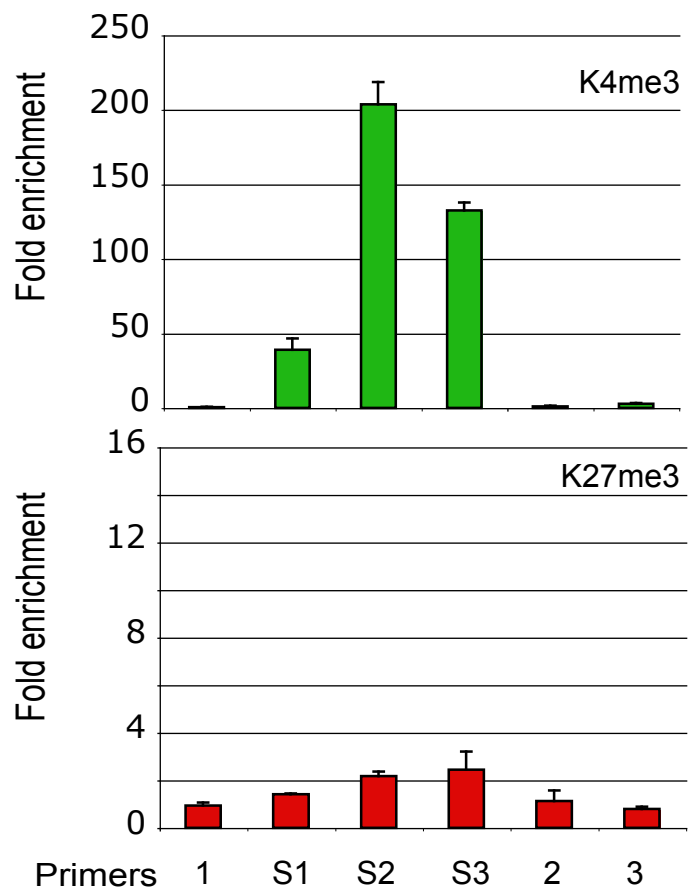

Supplement: Figure S8 — One additional mES cell clone containing gene desert BAC with the Sfxn2 CpG island was examined using ChIP-qPCR. As seen with the first clone (Figure 3B) this clone also shows significant enrichment of H3K4me3 but not H3K27me3 at the CpG island. Error Bars represent SEM (n = 2). (0.15 MB PDF) [file pgen.1001244.s008.pdf]

**A**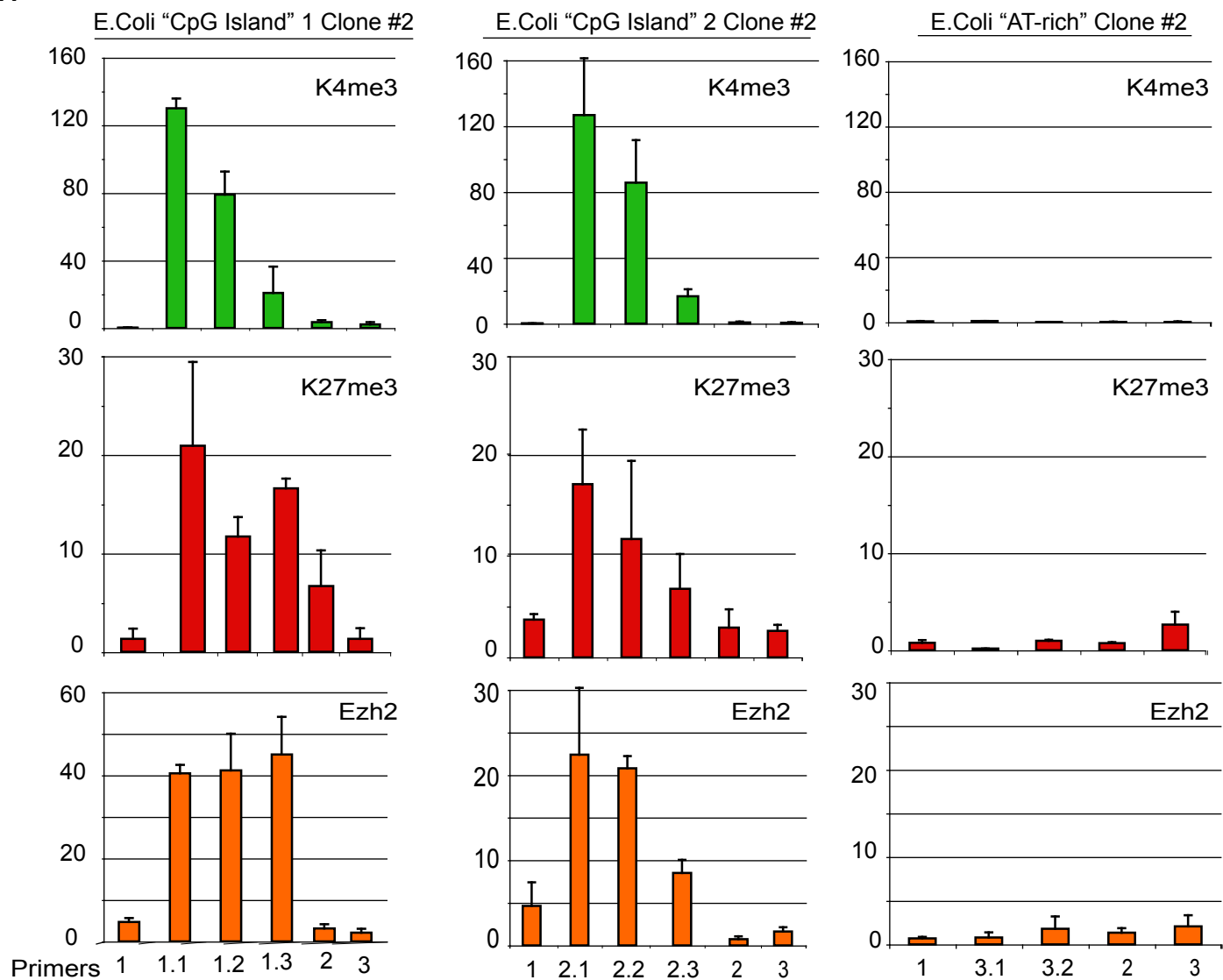**B**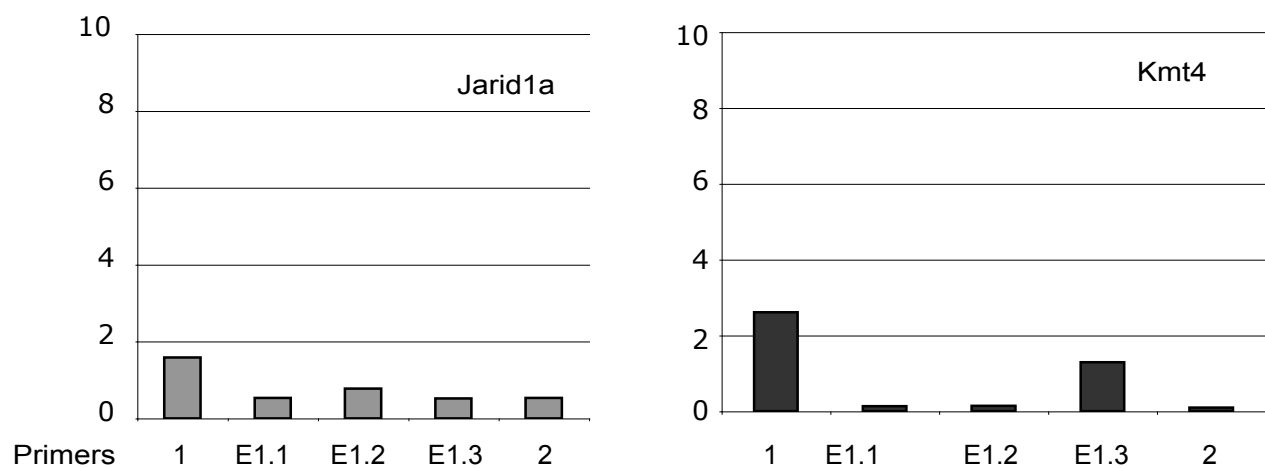

Supplement: Figure S9 — (A) One additional mES cell clone for each E. coli DNA construct was analyzed by ChIP-qPCR. As seen with the first clones (Figure 4A–4C) the CpG island clones show significant enrichment of K4me3, K27me3 and Ezh2 at the gene promoter. Error Bars represent SEM (n = 2) (B) As a negative control, E. Coli CpG island #1 was also tested for the chromatin modifiers Jarid1a and Kmt4, which showed no enrichment. (0.22 MB PDF) [file pgen.1001244.s009.pdf]

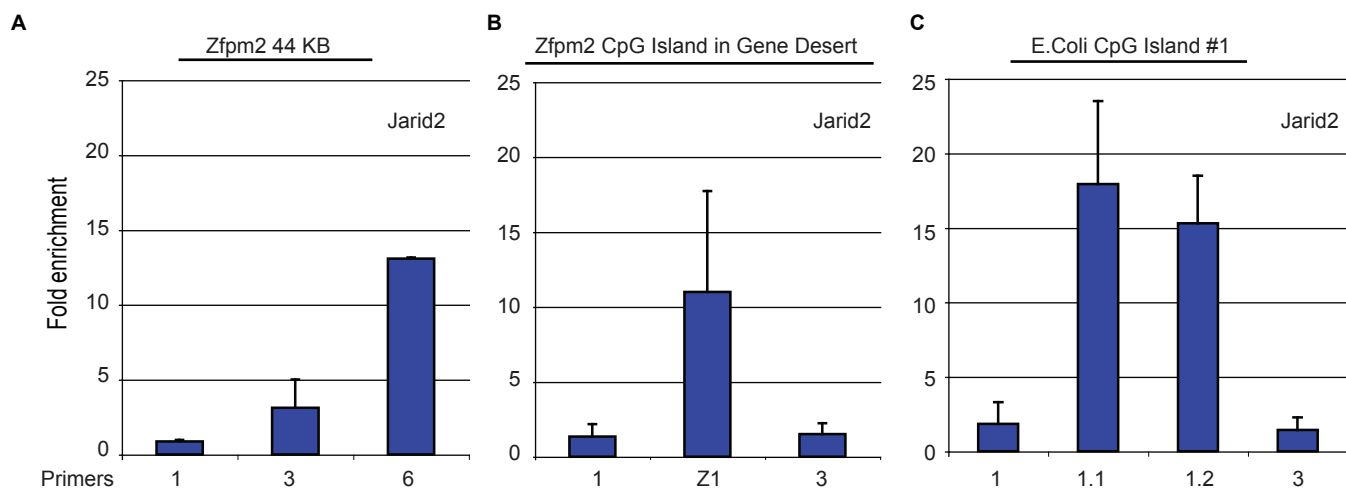

Supplement: Figure S10 — ChIP-qPCR shows Jarid2 enrichment signal at the CpG island (primer 6) of the 44 kb BAC (A), the Zfpm2 CpG island (primer Z1) within the Gene Desert BAC (B) and the GC-rich element (primers 1.1, 1.2) from E. Coli (C). Error Bars represent SEM (n = 2). (0.15 MB PDF) [file pgen.1001244.s010.pdf]
