# Supplementary material for: GC-Rich Sequence Elements Recruit PRC2 in Mammalian ES Cells
Source: PLoS Genet. 2010 Dec 9;6(12):e1001244. doi: 10.1371/journal.pgen.1001244 (PMC3000368; doi:10.1371/journal.pgen.1001244)
Supplement: Figure S4 — (A) Composite plots showing the lack of DNA methylation at both bivalent and K4me3 only promoters in mouse ES cells. (B) Schematic showing the CpG island of the Zfpm2 BAC remains free of DNA methylation upon integration into mouse ES cells. (C) The raw data used to create (B) shows aligned sequencing reads of Zfpm2 ES cell genomic DNA that was bisulfite treated (see Methods). Unmethylated and in vitro methylated BAC DNA are shown as controls. The underlined bases indicate sites of CG dinucletides. (0.22 MB PDF) [file pgen.1001244.s004.pdf]

A

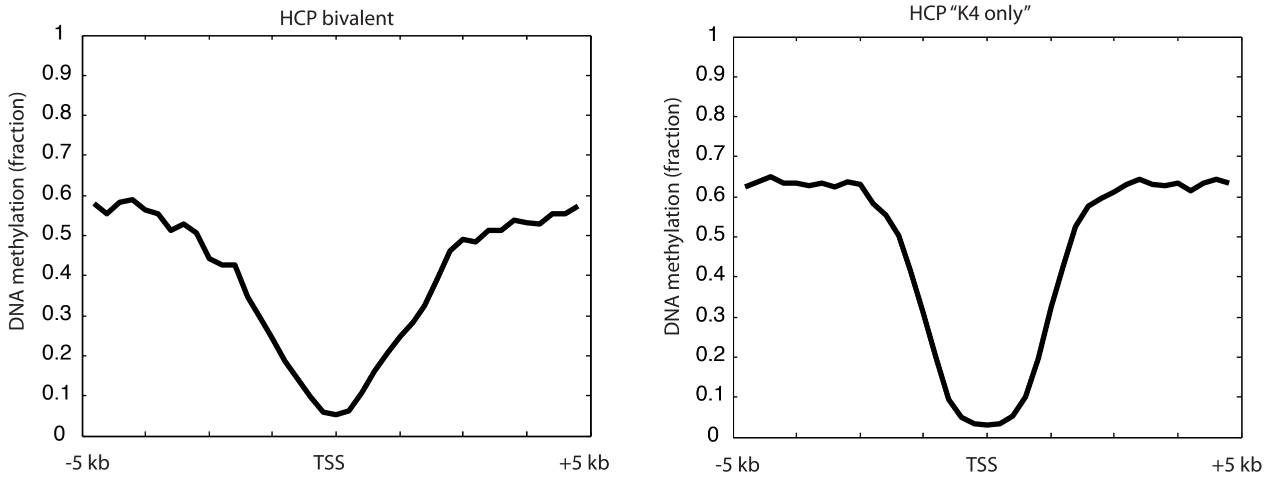

B

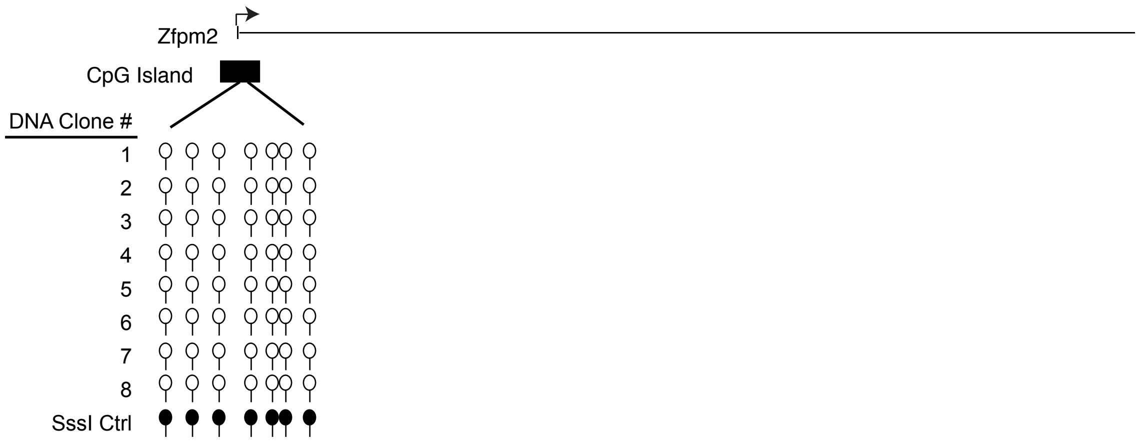

C

```
>BACcontrol:      GGGAGTGTATGGTTTTGAGTTTTTGGGTGTTTGGGGGTTTTGATGGTGTTTAGAGGATATGGTGGGTTAAGAGATATTTTTTGAAATAGGTATGTTTGTAGAGATTAAGTTGGTTAATTTTTTTTGGTTTGGGGAGAA
>BAC+Sss1Ctrl:    GGGAGTGTATGGTTTTGAGTTTTCGGGTGTTCGGGGGTTTCGATGGTGTTTAGAGGATATGGCGGGTTAAGAGATATTTTTCGAAATAGGTACGTTCGTAGAGATTAAGTCGGTTAATTTTTTTTGGTTTGGGGAGAA
>Rev1:            GGGAGTGTATGGTTTTGAGTTTTTGGGTGTTTGGGGGTTTTGATGGTGTTTAGAGGATATGGTGGGTTAAGAGATATTTTTTGAAATAGGTATGTTTGTAGAGATTAAGTTGGTTAATTTTTTTTGGTTTGGGGAGAA
>Rev2:            GGGAGTGTATGGTTTTGAGTTTTTGGGTGTTTGGGGGTTTTGATGGTGTTTAGAGGATATGGTGGGTTAAGAGATATTTTTTGAAATAGGTATGTTTGTAGAGATTAAGTTGGTTAATTTTTTTTGGTTTGGGGAGAA
>Rev3:            GGGAGTGTATGGTTTTGAGTTTTTGGGTGTTTGGGGGTTTTGATGGTGTTTAGAGGATATGGTGGGTTAAGAGATATTTTTTGAAATAGGTATGTTTGTAGAGATTAAGTTGGTTAATTTTTTTTGGTTTGGGGAGAA
>Rev4:            GGGAGTGTATGGTTTTGAGTTTTTGGGTGTTTGGGGGTTTTGATGGTGTTTAGAGGATATGGTGGGTTAAGAGATATTTTTTGAAATAGGTATGTTTGTAGAGATTAAGTTGGTTAATTTTTTTTGGTTTGGGGAGAA
>Rev5:            GGGAGTGTATGGTTTTGAGTTTTTGGGTGTCTGGGGGTTTTGATGGTGTTTAGAGGATATGGTGGGTTAAGAGATATTTTTTGAAATAGGTATGTTTGTAGAGATTAAGTTGGTTAATTTTTTTTGGTTTGGGGAGAA
>Rev6:            GGGAGTGTATGGTTTTGAGTTTTTGGGTGTTTGGGGGTTTTGATGGTGTTTAGAGGATATGGTGGGTTAAGAGATATTTTTTGAAATAGGTATGTTTGTAGAGATTAAGTTGGTTAATTTTTTTTGGTTTGGGGAGAA
>Rev7:            GGGAGTGTATGGTTTTGAGTTTTTGGGTGTTTGGGGGTTTTGATGGTGTTTAGAGGATATGGTGGGTTAAGAGATATTTTTTGAAATAGGTATGTTTGTAGAGATTAAGTTGGTTAATTTTTTTTGGTTTGGGGAGAA
>Rev8:            GGGAGTGTATGGTTTTGAGTTTTTGGGTGTTTGGGGGTTTTGATGGTGTTTAGAGGATATGGTGGGTTAAGAGATATTTTTTGAAATAGGTATGTTTGTAGAGATTAAGTTGGTTAATTTTTTTTGGTTTGGGGAGAA
>For1:            GGGAGNGTATGGTTTTGAGTTTTTGGGTGTTTGGGGGTTTTGATGGTGTTTAGAGGATATGGTGGGTTAAGAGATATTTTNTGAAATAGGTATNTTTGTAGAGATTAAGTTGGTTAATTTTTTTTGGTTTGGGGAGAN
>For2:            GGGAGTGTATGGTTTTGAGTTTTTGGGTGTTTGGGGGTTNTGATGGTGTTTAGAGGATATGGTGGGTTAAGAGATATTTTTTGAAATAGGTATGTTTGTAGAGATTAAGTTGGTTAATTTTTTTTGGTTTGGGGAGAA
>For3:            GGGAGNGTATGGTTTTGAGTTTTTGGGTGTTTGGGGGTTTTGATGGTGTTTAGAGGATATGGTGGGTTAAGAGATATTTTTTGAAATAGGTATNTTNGTAGAGATTAAGTTGGTTAATTTTTTTTGGTTTGGGGAGAA
>For4:            GGGAGNGTATGGTTTTGAGTTTTTGGGTGTTTGGGGGTTNNGATGGTGTTTAGAGGATATGGTGGGTTAAGAGATATTTTNNNAAATAGNNANNTTNGTAGAGATTAAGTTGGTTAATTTTTTTTNNTTTGGGGAGAA
```
